# Molecular Mechanisms and Therapeutic Potential of α- and β-Asarone in the Treatment of Neurological Disorders

**DOI:** 10.3390/antiox11020281

**Published:** 2022-01-29

**Authors:** Rengasamy Balakrishnan, Duk-Yeon Cho, In-Su Kim, Sang-Ho Seol, Dong-Kug Choi

**Affiliations:** 1 Department of Applied Life Science, Graduate School, BK21 Program, Konkuk University, Chungju 27478, Korea; balakonkuk@kku.ac.kr (R.B.); whejrdus10@kku.ac.kr (D.-Y.C.); 2 Department of Biotechnology, Research Institute of Inflammatory Disease (RID), College of Biomedical and Health Science, Konkuk University, Chungju 27478, Korea; kis5497@kku.ac.kr; 3 Research and Development, Sinil Pharmaceutical Co., Ltd., Seongnam-si 13207, Korea; seol@sinilpharm.com

**Keywords:** α-asarone, β-asarone, neuroprotection, neuroinflammation, molecular role, therapeutic, neurological disorders

## Abstract

Neurological disorders are important causes of morbidity and mortality around the world. The increasing prevalence of neurological disorders, associated with an aging population, has intensified the societal burden associated with these diseases, for which no effective treatment strategies currently exist. Therefore, the identification and development of novel therapeutic approaches, able to halt or reverse neuronal loss by targeting the underlying causal factors that lead to neurodegeneration and neuronal cell death, are urgently necessary. Plants and other natural products have been explored as sources of safe, naturally occurring secondary metabolites with potential neuroprotective properties. The secondary metabolites α- and β-asarone can be found in high levels in the rhizomes of the medicinal plant *Acorus calamus* (L.). α- and β-asarone exhibit multiple pharmacological properties including antioxidant, anti-inflammatory, antiapoptotic, anticancer, and neuroprotective effects. This paper aims to provide an overview of the current research on the therapeutic potential of α- and β-asarone in the treatment of neurological disorders, particularly neurodegenerative diseases such as Alzheimer’s disease (AD), Parkinson’s disease (PD), as well as cerebral ischemic disease, and epilepsy. Current research indicates that α- and β-asarone exert neuroprotective effects by mitigating oxidative stress, abnormal protein accumulation, neuroinflammation, neurotrophic factor deficit, and promoting neuronal cell survival, as well as activating various neuroprotective signalling pathways. Although the beneficial effects exerted by α- and β-asarone have been demonstrated through in vitro and in vivo animal studies, additional research is required to translate laboratory results into safe and effective therapies for patients with AD, PD, and other neurological and neurodegenerative diseases.

## 1. Introduction

The nervous system, a complex network of nerves and specialized cells, is responsible for the control of the body and communication among its parts. According to the World Health Organization (WHO), neurological disorders are defined as diseases of the central and peripheral nervous systems [1]. Neurological disorders can affect the brain, cranial nerves, peripheral nerves, and spinal cord and include neurotraumatic diseases, such as stroke and spinal cord injury; neurodegenerative diseases, such as Alzheimer’s disease (AD) and Parkinson’s disease (PD); as well as neuropsychological disorders, such as depression and schizophrenia [2]. In general, neurological disorders are characterized by acute and progressive neuron degeneration, ultimately resulting in brain dysfunction and neuronal cell death [3]. The underlying molecular mechanisms of neurodegeneration include alterations in phospholipid metabolism, accumulation of lipid peroxides, mitochondrial dysfunction, protein misfolding, abnormal protein aggregation, diminished cellular energy levels, disturbed calcium (Ca^2+^) homeostasis, excitotoxicity, oxidative stress, neuroinflammation, dysregulated hormonal signalling, and apoptosis [4,5,6].

AD, one of the most common neurodegenerative diseases, is characterised by the progressive worsening of learning, memory, and other cognitive functions with age. At the cellular level, AD is associated with the formation of extracellular plaques consisting of amyloid-beta (Aβ) and neurofibrillary tangles that lead to extensive neuronal loss. By reducing cellular energy levels and increasing oxidative stress, inflammation, and apoptosis, these “senile” plaques and aggregates lead to neuronal cell death [7,8,9].

PD, the second most common neurodegenerative disease, is characterized pathologically by the progressive loss of dopaminergic neurons in the substantia nigra pars compacta (SNpc) [10,11]. This nigral neuronal loss consequently results in dopamine (DA) deficiency in the striatum (ST), which is correlated with motor deficits such as tremors, rigidity, bradykinesia, postural instability, and gait impairment [12]. Dopaminergic cell death in PD is associated with the development of intracellular α-synuclein aggregates known as Lewy bodies [13].

Cerebral ischemic disease, a common form of stroke, is the fifth leading cause of death and disability impacting one million Americans every year [14]. It is caused by the blockage of blood vessels due to a thrombus or embolus [15]. At rest, the brain receives approximately 20% of the body’s total blood supply and is, therefore, highly sensitive to ischaemic events, and even short-lived ischaemia can result in significant cerebral damage [15]. During cerebral ischemia, part of the brain is deprived of oxygen and nutrients, which initiates a cascade of cellular and metabolic events that can result in severe brain damage. A considerable amount of evidence suggests that the release of excess glutamate during and after an ischemic insult leads to glutamate receptor hyperactivity, triggering harmful intracellular effects, including calcium overload and the generation of reactive oxygen species (ROS). This disruption of cellular homeostasis eventually leads to neurodegeneration [16,17]. 

Epilepsy is a neurological disorder characterized by temporary abnormal electrical activity in nerve cells [18]. In 2015, around 70 million people had been diagnosed with epilepsy worldwide, with 80% of these cases seen in developing countries [19]. Oxidative stress, glutamate excitotoxicity, and mitochondrial dysfunction, among others, have been implicated in the pathogenesis of epilepsy [20]. 

Several approaches have been proposed for the management of neuronal dysfunction and cell death associated with neurological disorders. However, current approaches primarily serve to reduce or manage symptoms, and no curative therapies have been introduced that can slow, prevent, or reverse disease development [21]. 

**Figure 1:**
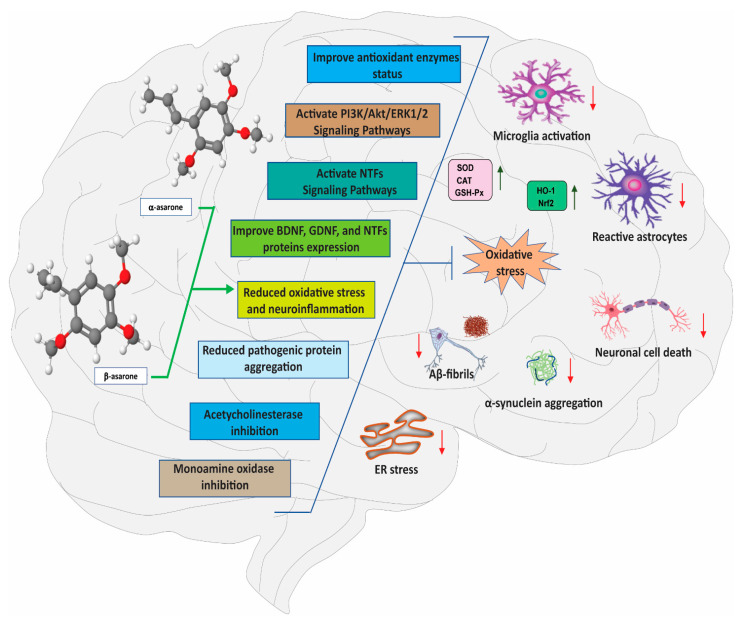
Molecular mechanism of neuroprotection by α- and β-asarone. The multi-target effects of α- and β-asarone in the brain include anti-oxidant, mitochondrial protecting, anti-apoptotic, anti-aggregation, anti-inflammatory, and the regulation of various neuroprotective signalling pathways. Red down-arrow (↓) and green up-arrow (↑) signs indicate inhibition and activation by α- and β-asarone treatment, respectively. BDNF, brain-derived neurotrophic factor; ERK, extracellular signal-regulated kinase; GDNF, glial cell-derived neurotrophic factor; PI3K/Akt, phosphatidylinositol-3-kinase; NTFs, neurotrophic factors.

Medicinal plants, found throughout the natural environment, represent an immense source of bioactive compounds in the form of secondary metabolites and other bioactive constituents [22]. Secondary metabolites derived from medicinal plants have been shown to exert beneficial effects on the chemical balance in the brain by influencing the function of several neurotransmitter receptors, with positive outcomes on cognitive disorders [23]. Recently, our research has identified α- and β-asarone, found in high levels in the rhizomes of the medicinal plant *Acorus calamus* (L.), as important secondary metabolites with potential therapeutic benefits for the treatment of AD, PD, and other neurological disorders. The mechanisms underlying α- and β-asarone-mediated neuroprotection are multipronged, and include antioxidant, antiapoptotic, anti-neuroinflammatory effects, as well as the modulation of various cellular and molecular targets; these actions might ultimately contribute to the ability of α- and β-asarone to attenuate the severity of neurological disorders [24,25,26,27] (Figure 1). Some of the specific molecular targets involved in α- and β-asarone-mediated neuroprotection have recently started to be unveiled. For instance, α- and β-asarone have been reported to promote disintegration of protein aggregates (tau, Aβ, and α–synuclein) associated with neurodegenerative disorders [28,29], attenuate lipopolysaccharide (LPS)-mediated neuroinflammation, promote neuronal cell survival, improve motor and non-motor functions, and prevent the neurodegeneration of dopaminergic neurons in the brain [24,25]. Antidepressant-like effects of α- and β-asarone have also been described [30], while Pan et al. [31] showed that β-asarone protected cortical neurons and reduced the infarction volume in an experimental model of ischemic stroke.

Here, we review recent research on the mechanisms by which α- and β-asarone exert neuroprotective effects in vitro and in vivo, in an effort to clarify their pharmacological properties and critically evaluate their potential as therapeutics for the treatment of neurological diseases.

## 2. Occurrence, Bioavailability, and Pharmacokinetics of α- and β-Asarone

The secondary metabolites α- and β-asarone ((E)-/(Z)-1,2,4-trimethoxy-5-prop-1-enylbenzene) are highly concentrated in the rhizomes of *Acorus calamus* Linn., *Acorus tatarinowii* Schott, and *Acorus gramineus* Solander, which belongs to the Acoraceae plant family (commonly known as “sweet flag”) [32]. α-asarone as an active phytochemical is also present in the bark of the Mexican tree *Guatteria gaumeri* Greenman from the *Annonaceae* family [33]. *A. calamus*, either alone or in combination with other herbs, has been extensively cultivated in various tropical and subtropical regions worldwide [32,34,35] and widely used as a traditional medicine for centuries [32]. *A. calamus* contains several phytoconstituents, including alkaloids, volatile oils, tannins, glycosides (xanthone), essential oils, flavonoids, monoterpenes, steroids, lignin, sesquiterpenes, saponins, mucilage, and polyphenolic compounds [36,37]. In the Ayurvedic system, *A. calamus* is extensively used to treat numerous inflammatory disorders [36,38,39,40], and in China, traditional practitioners prescribe *A. calamus* to treat constipation, digestive problems, and other health issues [41]. *A. calamus* and its primary bioactive constituents have also been found to reduce stress-induced immunosuppression in rats, resulting in improved immune function [42]. Both α- and β-asarone are widely studied bioactive secondary metabolites, featuring a broad range of pharmacological properties, including antioxidant, anti-inflammatory, neuroprotective, antidiabetic, anticancer, antifungal, antimicrobial, anti-ulcer, anti-allergic, wound healing, pesticidal, insecticidal, and radioprotective properties, among others [36,43,44,45,46,47].

### 2.1. Bioavailability and Pharmacokinetics of α- and β-Asarone

Due to their lipophilic character, α- and β-asarone have limited oral bioavailability, which can be improved by increasing their stability and solubility [48,49]. The plasma half-lives of α-and β-asarone are relatively short due to the rapid distribution of these agents to vital organs, such as the liver, spleen, heart, kidney, lungs, and brain [25,48,50,51]. Oral administration of the essential oil (single dose of 200 mg/kg) from *A. tatarinowii* Schott containing 11% α-asarone and 74% β-asarone to rats revealed that the maximum plasma concentrations were 0.5 μg/mL (*t_max_* = 11 min) for α-asarone and 2.5 μg/mL (*t_max_* = 14 min) for β-asarone, with half-lives in plasma of approximately 1 h [52]. Importantly, α- and β-asarone are distributed extensively throughout the brain, indicating their ability to permeate the blood–brain barrier (BBB), which is often a limiting factor when developing treatments for neurological disorders, including neurodegenerative diseases [24,25]. Lu et al. [49] also reported the rapid absorption and permeation of the BBB by α-asarone in rats. In another study, oral administration of α-asarone at 80 mg/kg resulted in 34% bioavailability [53]. A recent pharmacokinetic study demonstrated that the intravenous (i.v.) administration of lipid nanoparticles loaded with α-asarone resulted in significantly increased α-asarone levels detected in murine plasma and brain parenchyma fractions, compared with free α-asarone, confirming the ability to establish and maintain a therapeutic concentration of α-asarone in plasma that can be rapidly transported across the BBB [54]. In another study, the intranasal delivery of α-asarone to the brain using lactoferrin-modified methoxy poly(ethylene glycol)-poly(lactide) copolymer (mPEG-PLA) nanoparticles showed better BBB permeability without poor bioavailability, compared with i.v. administration. Intranasal α-asarone delivery enhanced the brain-targeting efficiency and reduced liver accumulation [55]. Another study demonstrated that the absolute bioavailability, brain-targeting efficiency, and percentage of nasal-mediated brain delivery of nasally administered PLA-α-asarone nanoparticles were 74.2%, 142.24%, and 29.83%, respectively, and nasal administration decreased drug-induced hepatotoxicity [56]. In an in vitro BBB model, borneol and α-asarone, used as co-adjuvant agents, improved the brain delivery of the central nervous system (CNS) drugs puerarin and tetramethylpyrazine. Because this effect could be counteracted by inhibitors of adenosine receptors, the authors concluded that α-asarone may gain entry to the CNS through adenosine receptors (AR), which represent an important pathway for drug delivery. Additionally, the co-administration of borneol and α-asarone decreased the expression of zonula occludens 1 (ZO-1), an important BBB junction protein, but increased A_1_AR and A_2A_AR expression. An in vivo pharmacokinetic analysis confirmed that the co-administration of borneol and α-asarone significantly increased the concentration of puerarin and tetramethylpyrazine in the brain, suggesting that a low dose of α-asarone not only improved the oral bioavailability of puerarin and tetramethylpyrazine but also increased BBB permeability, with α-asarone exhibiting superior permeability enhancement than borneol [57].

An absorption, distribution, metabolism, and excretion (ADME) in silico analysis revealed that β-asarone had good oral bioavailability and binding affinity towards dopaminergic receptors [58]. Furthermore, in silico results indicated that β-asarone was likely to interact with various amino acid residues in both the D2 and D3 dopamine receptors through hydrogen bonds [58]. In the same study, the toxicity of β-asarone was predicted using Lazar and ProTox, computational tools used to forecast the toxic properties of molecules. Lazar predicted that β-asarone is carcinogenic in various rodent models. Computational analysis of acute toxicity using ProTox showed that β-asarone had a high LD_50_ value (418 mg/kg) and the probability of the compound being mutagenic in *Salmonella typhimurium* was found to be 0.573 [58]. Another study reported that the half-lives of β-asarone in the cerebellum, thalamus, brainstem, cortex, hippocampus, and blood were 8.149, 2.832, 7.142, 1.937, 1.300, and 1.380 h, respectively [59].

Preliminary results from pharmacokinetic studies indicate the rapid and significant brain permeability of α- and β-asarone, expected to satisfactorily induce significant neuroprotective actions necessary to produce a beneficial therapeutic effect. However, further in vivo studies remain necessary to draw definitive conclusions regarding the ADME properties and overall safety of α- and β-asarone.

### 2.2. Toxicology of α- and β-Asarone: Preclinical Studies

Toxicity studies examining the effects of low doses of α- and β-asarone in rodent models have not revealed severe adverse effects. For instance, Chen et al. [60] reported that sub-chronic treatment with α-asarone (50 and 100 mg/kg, per os [p.o.], for 28 days) did not result in overt behavioural changes (walking, rearing, and grooming) in a seizure model generated in Swiss albino mice. However, α-asarone administered at a higher dose (200 mg/kg, p.o., for 28 days) significantly diminished spontaneous locomotor activity, although no mortality was observed. An acute toxicity test revealed that the oral median lethal dose (LD_50_) for α-asarone in mice was greater than 1000 mg/kg, with no deaths reported in any test groups [60]. In another study, mice were treated with α-asarone (150, 200, 250, 300, and 350 mg/kg) and survival was recorded for 14 days after treatment. The LD_50_ of α-asarone was calculated to be 245.2 mg/kg, with 95% confidence limits of 209.2–287.4 mg/kg. Deaths occurred mostly within 24 h after injection, and piloerection, ptosis, dyspnea, and ataxia were the most frequent clinical signs observed [61]. An in vivo subacute toxicity study revealed that the oral administration of β-asarone (100 mg/kg, for five consecutive days) reduced body weight and food consumption without causing mortality in pre-weanling rats [62]. Moreover, the weights of the adrenal glands and heart increased, the thymus weight decreased, and increased single-cell degenerative changes were observed in the thymus following β-asarone treatment. However, no significant changes in haematology or enzyme levels indicating hepatotoxicity were detected [62]. In yet another study, a long-term safety evaluation examining the effects of oral administration of β-asarone at 10 and 20 mg/kg p.o. for 90 days in mice did not reveal significant changes in any haematological parameters; however, blood concentrations of total bilirubin (BIL-T) increased following treatment with 20 and 50 mg/kg of β-asarone p.o. for 90 days; K^+^ concentrations decreased following treatments with 20 mg/kg/day of β-asarone p.o. for 90 days; and Cl^−^ concentrations decreased following treatments with 50 mg/kg of β-asarone p.o. for 90 days [63]. Following the oral administration of β-asarone at 200 µg/kg for 20 weeks in mice, no obvious toxicity was observed. During an LD_50_ toxicity study, treatment with β-asarone (500, 750, 1000, 1250, 1500, 1750, and 2000 mg/kg, i.v., for 24 h) did not result in marked behavioural changes and no obvious toxicity was observed [63]. Mice that died first appeared weak and less active, followed by gradual death, and the LD_50_ of β-asarone was calculated to be 1560 mg/kg [63]. Taken together, based on sub-acute toxicity tests, β-asarone at doses ≤100 mg/kg appears to be safe for clinical use, whereas the safety of doses > 100 mg/kg remains unclear. For further information on the toxicology of α- and β-asarone, we refer the reader to the following excellent reviews [27,64].

## 3. Neuroprotective Effects of α- and β-Asarone

In preclinical studies, α- and β-asarone show strong neuroprotective activities. At the molecular level, this neuroprotection has been attributed to antioxidant, anti-neuroinflammatory and antiapoptotic effects of α- and β-asarone, along with their ability to modulate various neuroprotective signalling pathways, such as the phosphatidylinositol-3-kinase (PI3K/Akt), cAMP-response element-binding protein (CREB), mitogen-activated protein kinase (MAPK), neurotrophic factors (NTFs), and Kelch-like ECH-associating protein 1 (Keap1)/nuclear factor-erythroid factor 2-related factor 2 (Nrf2)/antioxidant responsive element (ARE) axes. The neuroprotective effects of α- and β-asarone are summarized in Table 1 and the different pathways by which α- and β-asarone exert these effects are examined below.

### 3.1. Effects of α- and β-Asarone on Oxidative Stress

**Figure 2:**
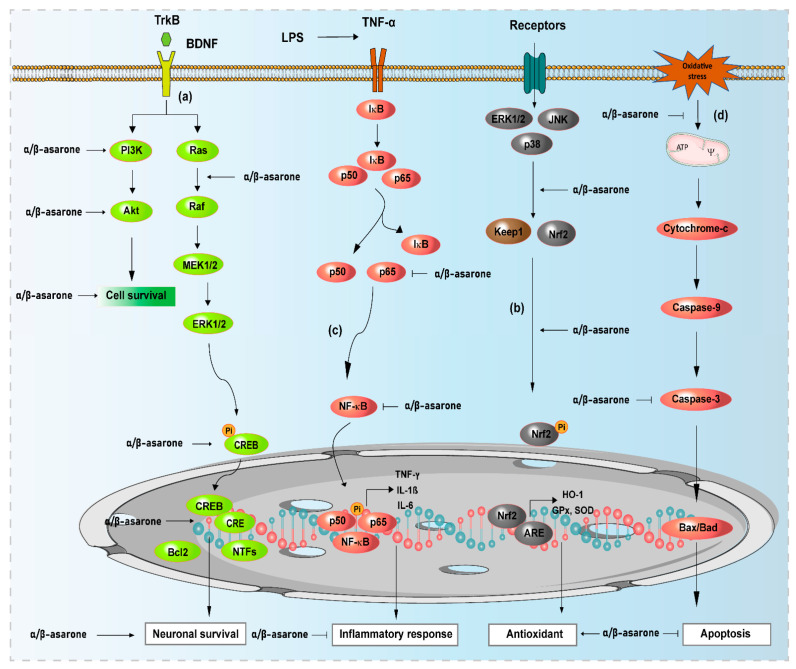
Neuroprotective effects of α- and β-asarone and their functional mechanisms and targets. (**a**) α- and β-asarone bind to receptors of neurotrophic factors (tropomyocin-related kinase; TrkB) and other binding proteins, activate downstream kinases (PI3K/Akt, ERK1/2) and phosphorylate CREB protein to promote neuronal survival; (**b**) α- and β-asarone activate Keap1/Nrf2/ARE signaling pathways to increase the expression of antioxidant enzymes, preventing oxidative stress by reducing cellular ROS levels; (**c**) α- and β-asarone block NF-κB activation, preventing the production of proinflammatory cytokines and other inflammation mediators, inhibiting neuroinflammation; (**d**) α- and β-asarone inhibit cytochrome C release from the mitochondria, mitigating the activation of caspases and Bax expression, and increasing Bcl2 expression, thus preventing apoptosis. The block lines (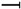) and arrows (→) indicate inhibition and activation by α- and β-asarone, respectively. ERK, extracellular signal-regulated kinase; Akt, protein kinase B; KEAP1, Kelch-like ECH-associated protein 1; Nrf2, nuclear factor erythroid 2-related factor 2; HO-1, heme oxygenase-1; ARE, antioxidant response element; CREB, cAMP-response element-binding protein; Bcl2, B-cell lymphoma 2; NF-κB, nuclear factor-kappa B; IκB, inhibitory kappa B; ERK1/2, extracellular signal-regulated kinases 1/2; NTFs, neurotrophic factors.

Oxidative stress results from an imbalance between the production of ROS and reactive nitrogen species (RNS) and the activity of antioxidant defence systems. Oxidative stress has been associated with the pathogenesis and progression of several neurodegenerative diseases, including AD and PD, and contributes to the damage associated with other neurological conditions (e.g., ischaemic stroke and schizophrenia) [65,66]. The antioxidant defence system neutralises the formation of excess free radicals in response to increased oxidative stress, preventing cellular damage. Various plant secondary metabolites with antioxidant properties exhibit demonstrable beneficial effects on brain function and overall health in humans [22,23]. Both in vitro and in vivo experiments have shown that α- and β-asarone exhibit antioxidant properties. The free-radical scavenging activities of α- and β-asarone have been demonstrated using various in vitro antioxidant assays, including the 2,2-diphenyl-1-picrylhydrazyl (DPPH) free radicals scavenging assay and the potassium ferricyanide reduction method, and by monitoring the levels of hydroxyl radicals, superoxide, and lipid peroxidation [67,68,69]. Interestingly, α- and β-asarone treatment significantly enhanced catalase (CAT), superoxide dismutase (SOD), and glutathione (GSH) activity levels and inhibited the excessive accumulation of malondialdehyde (MDA), lactate dehydrogenase (LDH), and ROS, suggesting that α- and β-asarone could improve enzymatic antioxidant defence systems [67,70,71]. Moreover, β-asarone pre-treatment was also found to activate the Nrf2 signalling pathway and its downstream target haemeoxygenase-1 (HO-1), which is involved in the quenching of ROS to mitigate oxidative stress [71]. When small interfering RNA (siRNA) was used to silence Nrf2, the protective effect of β-asarone was reduced, and H_2_O_2_-induced oxidative stress was enhanced in PC12 cells [71]. In addition, using an Aβ-stimulated PC12 cell model, Meng et al. observed that β-asarone pre-treatment could improve cell viability and mitigate cell damage and apoptosis. β-asarone could also decrease the level of ROS and MDA, increase the level of SOD, CAT, and GSH-PX, and promote the expression of Nrf2 and HO-1 [72] (Figure 2).

**Table 1:** Pre-clinical evidence supporting the neuroprotective effects of α- and β-asarone.

In Vitro/In Vivo	Study Model	Main Mechanism	Dose and Route of Administration	Reference
α-asarone				
BV-2	LPS/Parkinson’s disease (100 ng for 24 h)	↓ Microglial activation ↓ Neuroinflammation ↓ NF-κB activation	α-asarone (10, 50 and 250 µM., for 24 h)	[25]
PC12 and cultured rat astrocytes	tBHP/Dementia (0–300 μM., for 3 h)	↑ Neurotrophic factors ↑ Neurogenesis ↑ Akt activation ↑ Antioxidant response ↓ Oxidative stress ↓ PKA signalling ↓ Apoptosis	α- and β-asarone (15, 30 and 50 µM., 48 h)	[43,73,74]
Wistar rat	Noise stress/Stress model (100 dBA/4 h/d for 30 days)	↓ Oxidative stress ↓ AChE activity ↓ HSP70mRNA expression	α-asarone (9 mg/kg^−1^, i.p., 30 days)	[75]
ICR mouse	Scopolamine hydrochloride/Alzheimer’s disease (2 mg/kg, i.p., for 2 days)	↑ Motor performance ↓ Oxidative stress ↓ AChE activity	α-asarone (3, 10 and 30 mg/kg, p.o., 15 days)	[76]
C57BL/6 mice	Nicotine/Stress model (10–200 µg/mL for 40 days)	↑ Motor performance ↑ Neurotrophic factors ↑ p-CREB protein expression ↓ Weight loss	α-asarone (5, 10 and 20 mg/kg, i.p., 8 days)	[77]
APP/PS1 transgenic mice	Submicron emulsion injection/Alzheimer’s disease	↑ Motor performance ↑ Neuronal morphology ↑ Neuronal cell survival ↓ Neuroinflammation ↓ Aβ and tau aggregation ↓ Autophagosomes	α-asarone (30 and 60 mg/kg, i.p., 3 months)	[28]
Wistar rat	LPS/Neurotoxicity (30 µg/paw., for 6 h)	↑ Motor performance ↑ Cognitive function ↑ Anti-inflammatory ↑ Anti-nociceptive action ↓ Neuroinflammation ↓ LPS toxicity	α-asarone (3, 10 and 30 mg/kg, p.o., for 7 h)	[78]
C57BL/6 mice	MPTP/Parkinson’s disease (18 mg/kg, i.p., four injections at 2 h intervals for one day)	↑ Motor performance ↑ DA levels ↑ Cognitive function ↑ Anti-inflammatory ↑ TH-positive cells ↓ Neuroinflammation ↓ NF-κB activation	α-asarone (10 mg/kg, p.o., 15 days)	[25]
C57BL/6J mice	Ethanol/Dementia (Saline, i.p. + 2 g/kg ethanol, i.g., treatment duration not mentioned)	↑ Motor performance ↑ Cognitive function ↓ NMDA receptors ↓ SYNI activity ↓ Glu levels	α-asarone (7.5, 15 and 30 mg/kg, i.p., treatment duration not mentioned)	[79]
Wistar rat	Submicron emulsion injection/Alzheimer’s disease	↑ Motor performance ↑ Cognitive function ↑ Hippocampal neurons ↓ Aβ deposits	α-asarone (10 and 25 mg/kg, i.p., for 28 days)	[80]
**β-asarone**				
PC12	H_2_O_2_/Neurotoxicity (400 µM for 24 h)	↓ Oxidative stress ↓ ROS production ↑ Nrf2 and HO-1 activation	β-asarone (15, 30 and 60 µg/mL, for 24 h)	[71]
SH-SY5Y	Aβ/Alzheimer’s disease (SH-SY5Y, 20 μM for 24 h)	↓ Oxidative stress ↓ ROS production ↓ Apoptosis ↑ ASK1 siRNA activity	β-asarone (10–100 µg/mL, for 24 h)	[81]
SH-SY5Y	Aβ25-35/Alzheimer’s disease (20 μM for 24 h)	↓ Oxidative stress ↓ ROS production ↓ Neuroinflammation ↓ Apoptosis ↑ Autophagy efficiency ↑ Bcl2 protein expression	β-asarone (10, 50 and 100 µM, for 24 h)	[82]
Wistar rat	Middle cerebral artery occlusion (MCAO)/Ischemia	↑ Motor performance ↓ Oxidative stress	β-asarone (10, 20 and 30 mg/kg, p.o., for 30 days)	[83]
Wistar rat PC12 cells	MCAO/Ischemia OGD/R for 24 h	↑ Cell viability ↑ Motor performance ↓ Brain infarct volume ↓ Apoptosis ↓ Neuronal cell injury ↓ Neuroinflammation	α-asarone (10 and 20 mg/kg, i.v., for 24 h) α-asarone (12, 24, 48 μM for 24 h)	[84]
APP/PS1 transgenic mice	Alzheimer’s disease	↓ Senile plaques ↓ Aβ40 and Aβ42 aggregation ↓ Autophagosomes ↑ p62 expression	β-asarone (10, 20 and 40 mg/kg, i.g., for 30 days)	[85]
Wistar rat	6-OHDA/Parkinson’s disease (4 mg/mL, 6 µL in each rat for 30 days)	↑ DA levels ↑ TH-positive cells ↑ HSP70 expression ↑ p62 expression ↑ Neuronal cell survival ↓ α-synuclein ↓ Macroautophagy ↓ Autophagosomes	β-asarone (15 mg/kg, i.g., for 30 days)	[29]
C57BL/6 mice	MK-801/Schizophrenia (0.1 mg/kg, i.p., for 7 days)	↑ Motor performance ↑ Body weight ↑ Cognitive function ↑ Synaptophysin ↑ Postsynaptic density ↑ Cognitive function ↑ Anti-inflammatory ↓ Neuroinflammation ↓ Microglia activation	β-asarone (25 mg/kg, i.g., for 14 days)	[86]
Wistar rat	CUMS/Depression (CUMS for 21 days)	↑ Motor performance ↑ Body weight ↑ Cognitive function ↑ Neurogenesis ↑ BrdU-positive cells ↑ ERK1/2 activation ↑ CREB activation	β-asarone (25 mg/kg, i.g., for 28 days)	[87]
PC12	Aβ (1–42)/Alzheimer’s disease (20 μM for 24 h)	↑ Cell viability ↑ Bcl2 protein expression ↓ Apoptosis ↓ JNK signalling	β-asarone (7.5, 15, and 30 μg/mL, for 24 h)	[88]
Wistar rat	6-OHDA/Parkinson’s disease (4 µg/µL, 6 µL in each rat for 28 days)	↑ Motor performance ↑ DA levels ↑ TH-positive cells ↑ p62 expression ↑ Bcl2 expression ↓ α-synuclein ↓ Apoptosis ↓ JNK signalling	β-asarone (10, 20, 40 and 75 mg/kg, i.g., for 28 days)	[89]
AβPP/PS1 double-transgenic mice	Alzheimer’s disease	↑ Motor performance ↑ Cognitive function ↑ CREB activation ↑ Bcl2 expression ↑ CaMKII-α-positive cells ↓ Neuronal apoptosis	β-asarone (7 and 21 mg/kg, i.g., for 4 months)	[90]

↑ = increase, ↓ = decrease. i.p., intraperitoneal route; p.o., oral route; i.g., intragastrically; NF-κB, nuclear factor kappa B; PKA, protein kinase A; AChE, acetylcholinesterase; HSP70, heat shock protein 70; CREB, cAMP-response element-binding protein; p-CREB, phosphorylated CREB; Aβ, amyloid beta; LPS, lipopolysaccharides; DA, dopamine; TH, tyrosine hydroxylase; NMDA, N-methyl D-aspartate; ROS, reactive oxygen species; Nrf2, nuclear factor erythroid 2-related factor 2; HO-1, heme oxygenase-1; Bcl2, B-cell lymphoma 2; JNK, c-Jun N-terminal kinases; MPTP, 1-methyl-4-phenyl-1,2,3,6-tetrahydropyridine; 6-OHDA, 6-hydroxydopamine; CUMS, Chronic unpredictable mild stress; MCAO, middle cerebral artery occlusion; H_2_O_2_, hydrogen peroxide.

Recently, Pages et al. [70] and Saki et al. [91] reported an increase in the expression levels of the endogenous antioxidant enzymes SOD and glutathione peroxidase (GPx) in the brains of mice and rats treated with α- and β-asarone compared with untreated animals. Furthermore, α-asarone treatment attenuated the oxidative stress response in the brains of a rat model of noise-induced stress, an effect mediated by the increased expression of SOD, GPx, and CAT, in addition to the upregulation of other endogenous non-enzymatic antioxidant molecules, such as vitamin C, vitamin E, and GSH [92]. Similarly, α-asarone treatment increased acetylcholinesterase (AChE) activity and normalised MDA and SOD levels in the hippocampus and cerebral cortex of AD-like scopolamine-induced amnesic mice [76]. Another study reported that α-asarone administration attenuated brain and kidney damage induced by γ-radiation exposure by restoring antioxidant levels, such as SOD, GPx, CAT, and GSH, and decreasing the lipid peroxidation levels [93]. Yang et al. [83] observed that β-asarone supplementation significantly restored GSH, glutathione reductase (GR), CAT, and glutathione S transferase (GST) levels and decreased lipid peroxidation levels in the hippocampus of a rat model of ischaemia induced by middle cerebral artery occlusion (MCAO). Wang et al. [94] showed that β-asarone treatment could effectively attenuate MDA damage and significantly increase CAT and SOD activities in a rat model of AD generated by the intracerebroventricular injection of Aβ₁₋₄₂ combined with ischemia. In a rat model of MCAO-induced ischemic stroke, β-asarone treatment activated Nrf2/ARE pathway-related proteins, an effect that was inhibited by an Nrf2 inhibitor [31]. These findings suggest that the antioxidant effects of α- and β-asarone could contribute to their therapeutic benefits in the treatment of neurological disorders.

### 3.2. Effects of α- and β-Asarone on Neuroprotective Signaling Pathways

A variety of pro-survival signalling pathways, such as PI3K/Akt and extracellular signal-regulated kinase (ERK)1/2, are activated by α- and β-asarone (Figure 2) [72,73]. These pathways play an important role in cellular function, synaptic plasticity, and memory by altering the phosphorylation condition of molecules and modulating gene expression [95,96,97]. Oxidative stress triggers MAPK cascades that result in the activation of pro-survival p38 MAPKs, c-Jun N-terminal kinase (JNK), and ERK signalling pathways. The activation of ERK signalling suppresses the death-inducing complex formation and boosts cell survival by upregulating the anti-apoptotic proteins Bcl-xL and Bcl-2 and inhibiting Fas-mediated apoptosis [98].

In vitro results collected over the years have shown that α- and β-asarone activate the PI3K/Akt/Nrf2 and protein kinase A (PKA) signalling pathways, which play crucial roles in protecting the cells against abnormal ROS levels and neuronal damage, as well as improving cell viability and neuroprotective function [43,72,73]. In Aβ-treated PC12 cells, pre-treatment with β-asarone increased cell viability and decreased cell apoptosis [88]. The protective effect of β-asarone against β-amyloid-induced neurotoxicity was partly mediated through the inhibition of Aβ-induced JNK activation. Additionally, β-asarone significantly attenuated the Aβ-induced downregulation of Bcl-w and Bcl-xL and inhibited mitochondrial cytochrome C release and the activation of caspase-3 [88]. In primary cultured rat astrocytes and SH-SY5Y cells, α- and β-asarone upregulated Akt signalling and protected cells from oxidative stress, an effect that was shown to be mediated by the scavenging of ROS formation and the stimulation of the Nrf2-ARE self-defence mechanism, and the subsequent triggering of the expression of antioxidant enzymes, and increased the levels of the anti-apoptotic protein Bcl-2 [73,82]. Another recent study showed that β-asarone protects cells from Aβ_1–42_-induced cytotoxicity and attenuates autophagy via activation of the Akt-mTOR signalling pathway, which may be involved in the neuroprotection of β-asarone against Aβ toxicity in PC12 cells [99]. α- and β-asarone activate not only PI3K/Akt but also ERK/CREB/BDNF pathways in vivo and in vitro, which help to enhance memory function, protect neurons, and recover behavioural changes including immobility time, locomotor activity, and escape latency [77,90,100,101].

### 3.3. Effects of α- and β-Asarone on Proteostasis, ER Stress, and Autophagy

The accumulation of misfolded intracellular and extracellular protein aggregates is a major hallmark of neurodegenerative disease pathogenesis [102,103,104]. In the brain, several native proteins (Aβ, tau, and α-synuclein) undergo conformational changes due to genetic and environmental factors [104]. Neurodegeneration has been associated with mutated and misfolded protein monomers, oligomers, and insoluble aggregates, formed through altered β-sheet interactions, which cause dysfunction in cellular proteolytic pathways [105,106]. Pathogenic misfolded protein aggregation exerts neurotoxic effects on the CNS, resulting in eventual neuronal cell death and the development of neurodegenerative diseases [3,107]. Many therapeutic approaches to neurodegenerative diseases have aimed to diminish the accumulation of toxic oligomers, fibril deposits, and aggregation intermediates [108,109]. Several studies have suggested that some phytochemicals can inhibit amyloidogenic monomer synthesis and fibrillar aggregate formation, enhancing the formation of nontoxic aggregates and stimulating the proteolytic system to target the neurotoxic pathogenic factors associated with neuronal loss in neurodegenerative disease [110,111].

**Figure 3:**
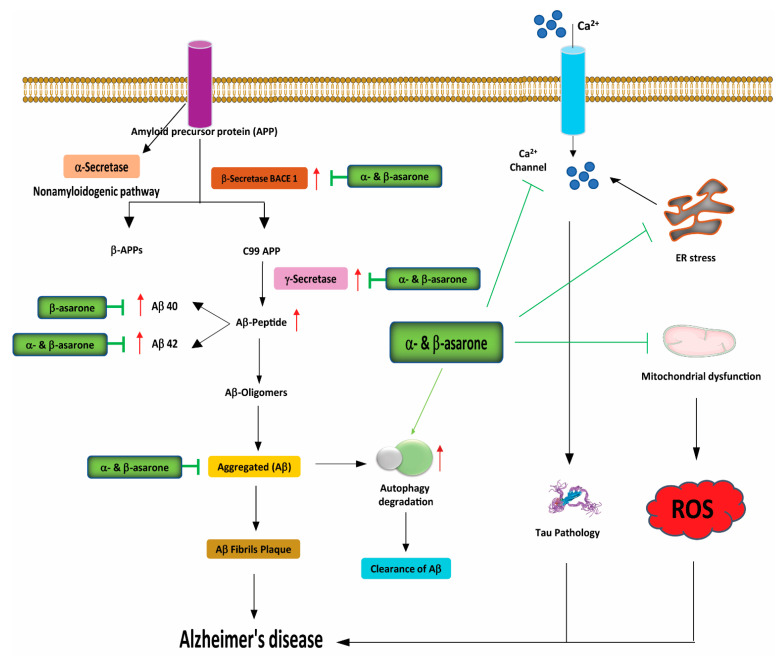
Neuroprotective effects of α- and β-asarone on the pathogenic mechanisms of Alzheimer’s disease (AD). The 99 amino acid C-terminal fragment of amyloid precursor protein APP-C99 (C99) is cleaved by γ-secretase to form the Aβ peptide, which plays a critical role in the etiology of AD. The 40-residue peptide Aβ (1–40) represents the most abundant Aβ isoform in the brain, while the 42-residue Aβ (1–42) shows a significant increase with certain forms of AD. α- and β-asarone block the formation of Aβ peptides by reducing γ-secretase, and lessen tau pathology by blocking intracellular calcium channels and inhibiting oxidative stress-mediated neuronal cell death by decreasing ROS release from the mitochondria and promoting the autophagic clearance of aggregate-prone proteins. Aβ, amyloid beta; APP, amyloid precursor protein; ROS, reactive oxygen species; ER stress, endoplasmic reticulum stress.

Amyloidogenic Aβ_1–42_ peptides are primarily generated by the cleavage of amyloid precursor protein (APP) by β- and γ-secretase [112]. Recent studies have found that α- and β-asarone suppress the expression of the β-secretase BACE1, improving cognitive and behavioural function in animal models of AD [113,114]. A very recent study found that α-asarone potentially targets the Aβ and tau pathology pathways by inhibiting Aβ_42_ aggregation, in addition to inhibiting tau phosphorylation, resulting in improved spatial learning memory in APP/presenilin-1 (PS1) transgenic mice [28] (Figure 3). Another study demonstrated that treatment with β-asarone reduced the number of senile plaques and decreased Aβ_40_, Aβ_42_, and APP expression levels in the hippocampus of APP/PS1 transgenic mice [85]. Moreover, β-asarone displayed a significant therapeutic effect against toxic protein deposition and increased the expression of the presynaptic protein synapsin 1 (SYN1), which should remove toxic superoxide anion radicals produced in cells [114]. A thioflavin T (ThT) fluorescence assay conducted in PC12 cells treated with α- and β-asarone showed an efficient and dose-dependent reduction in Aβ aggregation, protecting PC12 cells from Aβ aggregate-induced toxicity [115] (Figure 3).

**Figure 4:**
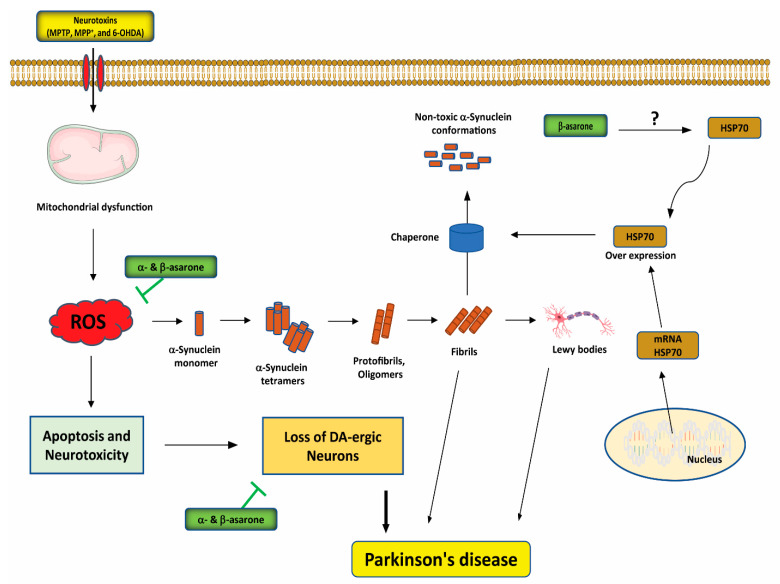
Neuroprotective effects of α- and β-asarone on the pathogenic mechanisms of Parkinson’s disease (PD). α- and β-asarone inhibit oxidative stress-mediated neuronal death and attenuate ROS release from the mitochondria. By inducing HSP70, β-asarone may potentially attenuate/or prevent protein misfolding, reducing the apoptosis of DA neurons. The question mark (?) indicates that the mechanisms by which α- and β-asarone interact with HSP70 to exert neuroprotective effects in PD are unclear. MPTP, 1-methyl-4-phenyl-1,2,3,6-tetrahydropyridine; MPP^+^, 1-methyl-4-phenylpyridinium; 6-OHDA, 6-hydroxydopamine; PD, Parkinson’s disease; DA, dopamine; ROS, reactive oxygen species; HSP70, heat shock protein 70.

Presynaptic α-synuclein aggregation is considered the primary pathogenic factor in the development of α-synucleinopathies, such as PD [103]. Large α-synuclein aggregates, associated with the presence of missense mutations in the α-synuclein gene, including A30P, A53T, and E46K, may exert toxic effects through increased oxidative stress, mitochondrial dysfunction, altered patterns of phosphorylation, and interactions with the phospholipid membrane [116]. Moloney et al. [117] demonstrated that heat shock protein 70 (HSP70) protects dopaminergic neurons from protein aggregation and inhibits microglial activation, ultimately preventing apoptosis. The overexpression of HSP70 exerts a protective effect against early-onset α-synuclein–induced pathology, as demonstrated in an adeno-associated virus model of α-synuclein aggregation [118]. β-asarone treatment has also been found to alleviate dopaminergic cell death induced by 6-hydroxydopamine (6-OHDA) through the upregulation of HSP70 mRNA and protein expression levels and the downregulation of α-synuclein mRNA and protein expression levels [29] (Figure 4). In addition, in SH-SY5Y cells transfected with α-synuclein, β-asarone treatment protected against cell death induced by MPP^+^ [119]. In a mouse model, β-asarone treatment also exerted neuroprotective effects against 1-methyl-4-phenyl-1,2,3,6-tetrahydropyridine (MPTP)-induced PD, preventing the typical decrease in tyrosine hydroxylase (TH)-positive cells and increasing α-synuclein expression levels, thus protecting dopaminergic neurons in the midbrains [119] (Figure 4).

Pathogenic proteins associated with neurodegenerative diseases cause dysfunction in the cellular proteolytic systems, including the ubiquitin-proteasome system (UPS) and the autophagy–lysosome pathway [120]. Misfolded and unfolded proteins are identified by ubiquitination and targeted for degradation by the proteasome, and UPS dysfunction has been associated with the aggregation of misfolded proteins in PD, although the specific role played by the UPS in PD pathogenesis remains unclear [121]. Disease-specific protein aggregates that are too large for the UPS, including Aβ, tau, and α-synuclein aggregates, in addition to damaged organelles, such as mitochondria, are typically targeted for degradation by the autophagy–lysosome pathway [105].

The endoplasmic reticulum (ER) plays a critical role in the proper folding of membrane-bound and non-cytoplasmic proteins [122,123,124]. The accumulation of misfolded or unfolded proteins in the ER causes cellular stress and triggers the unfolded protein response (UPR). Chronic or excessive UPR activation can eventually lead to cell death. UPR activation is mediated by three ER stress sensors: protein kinase RNA-like endoplasmic reticulum kinase (PERK), inositol-requiring enzyme-1 (IRE1), and activating transcription factor 6 (ATF6) [122,123,124]. The glucose-regulated protein GRP78 (also, known as BiP) primarily regulates the initiation of the UPR through its direct interactions with each signal-transducing sensor [122,125]. Like ATF6, ATF4 is a basic leucine zipper (bZIP) transcription factor important for maintaining intracellular homeostasis through the upregulation of UPR-target genes involved in efficient protein folding, the antioxidant response, and amino acid biosynthesis and transport. In addition to promoting an adaptive response, ATF4 upregulates the bZIP transcription factor C/EBP homologous protein (CHOP), which promotes cell death [122,126]. Collectively, the UPR pathways serve to halt protein biosynthesis, promote protein degradation, and generate chaperones to refold misfolded proteins.

**Figure 5:**
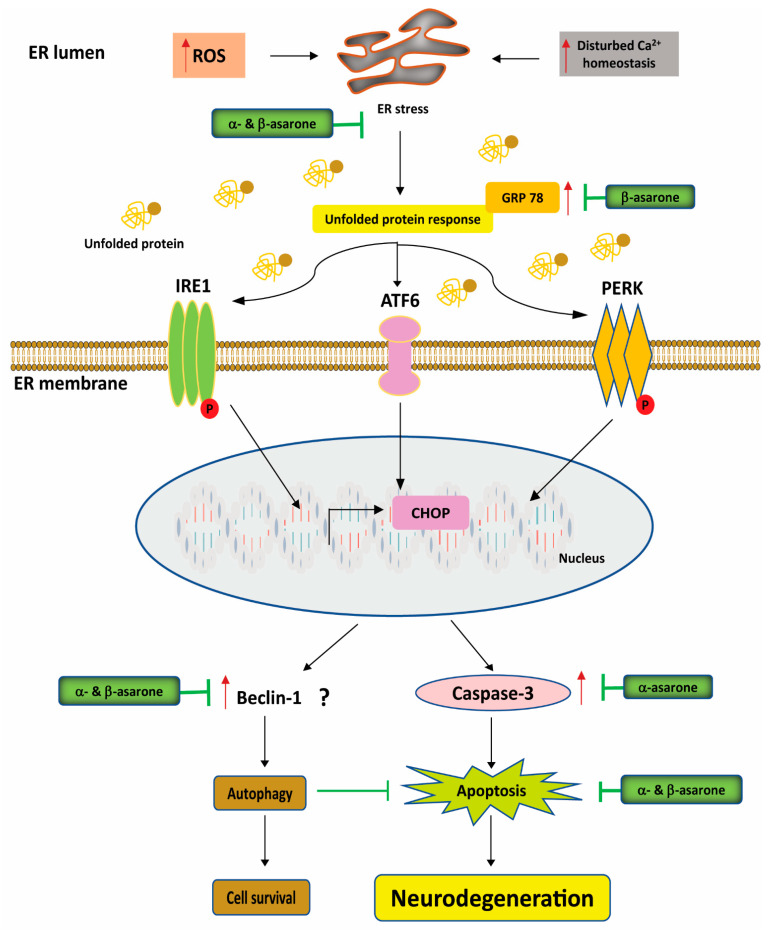
Neuroprotective effects of α- and β-asarone against neurological disease-associated ER stress. ER stress is detected by three UPR activator proteins, IRE1, PERK, and ATF. β-asarone inhibits the ER stress proteins GRP78 and CHOP, blocking two of the three UPR pathways. α- and β-asarone may also inhibit Beclin-1 expression, which promotes autophagy activation. The question mark (?) indicates that the mechanism by which α- and β-asarone affect Beclin-1 is still unclear. ROS, reactive oxygen species; ER stress, endoplasmic reticulum stress; GRP78, glucose-regulating protein 78; 6-OHDA, 6-hydroxydopamine; CHOP, C/EBP homologous binding protein; ATF6, activating transcription factor 6; IRE1, inositol-requiring enzyme 1; PERK, PERK phosphorylation.

ER stress has been identified in several experimental cellular models of PD [127], and the increased expression of wild-type α-synuclein is sufficient to cause ER stress [128]. ER stress is closely related to oxidative stress, which can also trigger UPR activation [129]. In a 6-OHDA-induced PD model, β-asarone treatment downregulated the mRNA levels of GRP78 and CHOP, resulting in the blockade of two of the three UPR activation pathways [130] (Figure 5). GRP78 preferentially binds unfolded or misfolded proteins in the ER, releasing its inhibitory hold on PERK, ATF6, and IRE1 [131]. Furthermore, β-asarone treatment was shown to reduce the expression of GRP78, phosphorylated PERK (p-PERK), and CHOP, regulating ER stress response and autophagy in a 6-OHDA-induced rat model of PD [132] (Figure 5). Interestingly, α-asarone treatment prevented 7-hydroxycholesterol-triggered macrophage apoptosis by alleviating ER stress-specific signalling, such as caspase induction and CHOP activation [133]. Furthermore, α-asarone treatment significantly reduced chronic constriction injury-induced ER stress in the spinal cord, in addition to decreasing microglial activation, and alleviating neuropathic pain [134]. In a recent in vitro study, pre-treatment with α-asarone protected hippocampal cells from oxidative and ER stress by decreasing ROS production and suppressing PERK signalling in glutamate- and tunicamycin-induced hippocampal HT22 cells [135].

Autophagy activation can upregulate the clearance of protein aggregates, prevent mitochondrial damage, control axon homeostasis and neurogenesis, ensure cell survival, and reduce growth factor deficiency and ER stress, with potential therapeutic benefits in slowing the pathological progression of AD, PD, and other neurodegenerative diseases [136,137]. Beclin-1, phosphorylated (p)-Akt, and mammalian target of rapamycin (mTOR) are known autophagy regulators, and some plant-derived secondary metabolites have been shown to exert neuroprotective effects via the stimulation of autophagy through both mTOR-dependent and independent mechanisms [138]. In a 6-OHDA-induced PD mouse model, β-asarone treatment significantly decreased the levels of both mRNA and protein of Beclin-1 and LC3B, and increased p62 expression, indicating autophagy activation [29]. Another study found that treatment with α-asarone enhanced autophagy in macrophages by upregulating autophagolysosomal formation [139]. A recent canonical correlation analysis revealed that β-asarone treatment can inhibit Aβ aggregation through the promotion of autophagy in a PC12 cell model of AD [114]. Another experiment demonstrated that α-asarone reduced the activation of eIF2α-CHOP by 7β-hydroxycholesterol, enhanced autophagy in macrophages through the upregulation of autophagolysosomal formation, increased phosphorylation of Bcl-2, and facilitated the entry of Beclin-1 into the autophagic process [139]. These findings suggest that α- and β-asarone may have the potential to reduce protein aggregation and ER stress through autophagy modulation, which can have regulatory effects on neurodegenerative disease progression.

### 3.4. Effects of α- and β-Asarone on Neuroinflammation

Microglia are located throughout the CNS and play important roles in the clearance of damaged neurons, infection prevention, foreign organism defence, and tissue repair [140]. Microglial activation is the main initiator of neuroinflammatory responses, and microglia can be activated by the presence of pathogens, tissue damage, infection, injury, or neurotoxins. Microglial activation leads to the release of various oxidants, including ROS and RNS, in addition to the induction of inflammatory factors, such as pro-inflammatory cytokines, chemokines, and neurotoxic molecules, triggering neuroinflammatory responses that contribute to neuronal cell death and promote the progression of neurodegeneration [141,142]. Consequently, the inhibition of pro-inflammatory cytokine release by microglial activation has been explored as an effective therapeutic intervention for attenuating neurological disorders [143,144,145].

Plant-derived secondary metabolites with strong antioxidant capacities can reduce the prevalence of age-related neurological disorders associated with increased inflammation [146,147]. In vitro and in vivo experiments have shown that, by attenuating the expression of pro-inflammatory cytokines and inflammatory mediators, including tumour necrosis factor (TNF)-α, interleukin (IL)-6, and IL-1β, preventing inflammasome formation, or inducing the expression of neuroprotective anti-inflammatory and antioxidant molecules, α- and β-asarone can exert neuroprotective effects in various neurological disorders [78,82] (Figure 2). For instance, in the pilocarpine-induced rat model of epilepsy, α-asarone treatment attenuated cognitive deficits and reduced neuroinflammation and microglial activation via a reduction in NF-κB activation [148]. Treatment with α-asarone also suppressed pro-inflammatory cytokine production and attenuated the LPS-stimulated neuroinflammatory response in primary cultured microglial cells. Treatment with α-asarone further inhibited the activation of the inflammatory response by significantly reducing the degradation of the NF-κB inhibitors IκB-α and IκB-β and regulating NF-κB transcription in both in vitro primary cultured microglial cells and an in vivo pilocarpine-induced epileptic rat model [148] (Figure 2). In the MPTP-induced mouse model of PD, α-asarone treatment significantly reduced microglial activation and neuroinflammation in the brain [25]. In vitro studies have revealed that α-asarone treatment significantly attenuates the LPS-stimulated upregulation of neuroinflammatory responses, reduces pro-inflammatory cytokine production, and inhibits NF-κB activation by blocking the degradation of NF-κB inhibitor IκB-α in BV-2 microglial cells [25] (Figure 2). In the Aβ_1-42_-induced APP/PS1 transgenic mouse model of AD, the oral administration of α-asarone significantly decreased pro-inflammatory cytokine levels and reduced the expression of the microglial marker protein glial fibrillary acidic protein, alleviating neuroinflammation [28]. Similarly, in a rat model of a spinal cord injury, the oral administration of α-asarone significantly reduced the levels of IL-1β, IL-6, TNF-α, inducible nitric oxide synthase, monocyte chemoattractant protein 1 (MCP-1), and macrophage inflammatory protein 2, whereas the levels of anti-inflammatory mediators IL-4, IL-10, and arginase 1 were increased 24 h after spinal cord injury [149]. In addition, immunohistochemistry analysis revealed that α-asarone treatment effectively reduced neuroinflammation and reactive gliosis and increased the expression of M2 macrophage markers and angiogenesis [149].

In the LPS-stimulated BV-2 cell model, treatment with α-asarone decreased the mRNA and protein levels of MCP-1, modulated microglial morphological dynamics, and reduced the numbers of activated microglia and microglial process tips while promoting neurogenesis [150]. In a mouse model of chronic schizophrenia induced by MK-801, β-asarone treatment improved cognitive functions and synaptic plasticity, partly by suppressing microglial activation and downregulating the microglia-mediated inflammatory response [86]. Likewise, in an in vitro study, β-asarone treatment in Aβ_1–42_-induced PC12 cells reduced IL-1β and TNF-α levels, inhibited NF-κB activity, and downregulated the phosphorylation of extracellular signal-regulated kinase (ERK), p38, and c-Jun N-terminal kinase (JNK) [100].

Taken together, a substantial amount of in vitro and in vivo data highlight the potential of α- and β-asarone as promising therapeutic agents against neurological disorders due to their antioxidant and anti-inflammatory properties, and their ability to downregulate the NF-κB pathway.

### 3.5. Effects of α- and β-Asarone on Neurogenesis, Neurotransmitter Metabolism, and Neuronal Cell Death

Neurotrophic factors (NTFs), such as the brain-derived neurotrophic factor (BDNF) and the glial-cell-derived neurotrophic factor (GDNF), play central roles in the survival, growth, functional development, and plasticity of glutamatergic and γ-aminobutyric acid (GABA)ergic synapses. NTFs modulate neuronal differentiation, influencing serotonergic and dopaminergic neurotransmission [151,152,153]. Accordingly, BDNF and GDNF have been identified as potential therapeutic targets in neurodegenerative diseases, and pre-clinical studies have reported the beneficial effects of the administration of BDNF and GDNF in PD models [154,155,156,157,158]. However, these essential NTFs cannot pass through the BBB, preventing NTFs directly administered to patients from reaching damaged neurons in the brain. Plant-derived secondary metabolites that are able to cross the BBB can directly target these same neurotrophic receptors, potentially activating the neuronal biogenesis of BDNF and GDNF [98,159].

β-asarone has been reported to enhance the levels of BDNF-targeted receptor-like tropomyosin-related kinase B (TrkB) and activate the ERK pathway, triggering antidepressant-like effects, enhancing the survival rate of cultured motor neurons, and significantly reducing the apoptosis rate of hippocampal neurons [160]. Interestingly, in cultured rat astrocytes and PC12 cells, α- and β-asarone treatment significantly promoted the expression and secretion of NTFs, such as nerve growth factor (NGF), BDNF, and GDNF, in a dose-dependent manner [43,74]. In addition, the application of the protein kinase A (PKA) inhibitor H89 to cultured astrocytes partially blocked α- and β-asarone-induced NTF expression, suggesting the involvement of PKA signalling in this effect [43] (Figure 2). Moreover, β-asarone treatment induced the expression of BDNF and GDNF and exerted neuroprotective activities via the ERK/cAMP signalling pathways in both in vitro and in vivo models [100,161]. Monoamine oxidase (MAO), an enzyme located in the outer mitochondrial membrane, is principally responsible for monoamine neurotransmitter homeostasis (epinephrine, norepinephrine, serotonin, and DA) in the brain, and regulates signalling pathways involved in neuron survival and death [162]. MAO exists in two forms, termed MAO-A and MAO-B. MAO-A inhibitors have anxiolytic and antidepressant activities, whereas MAO-B inhibitors, such as rasagiline and selegiline, activate neuroprotective pathways [163]. β-asarone and levodopa co-administration were found to decrease MAO-B activity, enhancing monoaminergic neurotransmitter levels [164].

The beneficial effects of α- and β-asarone on dopaminergic neuronal loss have been associated with increased DA levels, both in vitro and in vivo. In addition to the various potential mechanisms that contribute to the prevention of dopaminergic cell death, as described above, the co-administration of β-asarone and levodopa (L-DOPA) has been shown to increase the striatal and plasma levels of DA and DA metabolites, including 3,4-dihydroxyphenylacetic acid (DOPAC), homovanillic acid (HVA), and 5-hydroxytryptamine (5-HT). Elevated DA metabolites were also observed in cortical and hippocampal regions [164,165,166]. Increased DA levels were associated with improved behavioural performance of 6-OHDA-induced rat models of PD [164]. Similarly, α-asarone treatment has been shown to increase the levels of the DA metabolite DOPAC in the striatum of an MPTP-induced mouse model of PD [25]. Treatment with β-asarone in 6-OHDA-induced rats resulted in significantly increased levels of DOPAC, HVA, and the serotonin metabolite 5-hydroxyindoleacetic acid in the striatum, accompanied by improved motor performance [89].

β-asarone treatment was also found to attenuate learning and memory impairments in both rat pups and adult rats in a chronic lead (Pb)-induced model of developmental delay, partially rescuing learning and memory abilities [167]. Interestingly, a previous study suggested that changes in the levels of amino acids in AD, particularly the imbalance between glutamate and GABA levels, are key factors contributing to neuronal injury and memory impairment [168,169]. Moreover, in an ethanol-induced dementia mouse model, the administration of α-asarone was able to maintain the equilibrium between glutamate and GABA in the hippocampus, enhancing learning and memory abilities [79]. As a result, in addition to potential disease-modifying properties, α- and β-asarone may also prove useful for symptom relief in neurological disorders.

Cell death by apoptosis is an important mechanism of cell loss in neurodegenerative diseases. Apoptosis occurs in response to a variety of biochemical and morphological alterations, including cell shrinkage, nucleosomal degradation, and chromatin condensation. Apoptosis signalling by mitochondria is considered a promising therapeutic target for the treatment of neurodegenerative disease [170,171]. The first evidence of α- and β-asarone-mediated protection against neuronal cell death was deduced from in vitro studies using NMDA-treated primary cultured rat cortical cells and Aβ-treated PC-12 cells as models of neurodegeneration [172,173]. The oral administration of α-asarone to an MCAO-induced mouse model of ischaemic stroke significantly increased the number of neuronal nuclei (NeuN)-immunoreactive cells and decreased the number of S100 calcium-binding protein β (S100β)-immunoreactive cells, resulting in greater-than-additive effects on sensorimotor function and motor balance, based on analyses of corner and rotarod behavioural tests [26]. A similar study showed that α-asarone at a dose of 10 and 20 mg/kg treatment could decrease brain infarct volume, improve neurological function, and reduce the incidence of post-stroke epilepsy [84]. These neuroprotective effects were attributed to improved glial activation and autophagy, indicating that α-asarone is a very promising drug candidate for cerebral ischemia stroke [84]. In an MPTP-injected mouse model of PD, α-asarone treatment suppressed glial activation, protected dopaminergic TH-positive neurons in the substantia nigra and TH-positive fibres in the striatum, and attenuated PD-like behavioural impairments, as assessed by the Y-maze and pole tests [25]. Another experiment showed that α-asarone significantly improved behavioural performance and learning and memory in C57BL/6J mice treated with ethanol to induce cognitive impairment [79]. Moreover, treatment with β-asarone partially reversed chronic unpredictable mild stress (CUMS)-induced depression-like behaviours, as assessed by both the sucrose preference and forced swim tests, which was associated with increased hippocampal neurogenesis, as indicated by bromodeoxyuridine (BrdU) immunoreactivity [87]. α-asarone treatment significantly attenuated the immobility time of mice subjected to nicotine withdrawal, as assessed by the forced swim test, indicating an antidepressant effect of α-asarone, with no apparent effect on locomotor activity [77]. In immunostaining and microscopy analyses following chronic α-asarone treatment, both the size and morphology of hippocampal neurons returned to a relatively normal state [80]. In aged rats, treatment with α-asarone also enhanced cognitive functions, an effect that was correlated with a reduction in neuronal loss and decreased toxic Aβ production [80].

An increasing body of evidence has shown that Bcl-2, along with Bax and caspase proteins, play major roles in the apoptotic pathways associated with the pathogenesis of neurodegenerative diseases [174,175,176]. The downregulation of the anti-apoptotic regulators Bcl-2, Bcl-xL and Bcl-w results in the inhibition of JNK phosphorylation, the release of cytochrome c from the mitochondria, and caspase-3 activation, ultimately leading to apoptosis. β-asarone treatment prevented the downregulation of these anti-apoptotic proteins, reducing apoptosis in neuronal PC-12 cells and ameliorating Aβ-induced cognitive impairments in the hippocampus of an Aβ-induced rat model of AD [88,177]. A recent study showed that β-asarone treatment protected against 6-OHDA-induced dopaminergic neurodegeneration in a rat model of PD through the downregulation of JNK and p-JNK expression, resulting in the indirect increase in Bcl-2, accompanied by improved behavioural performance in the open-field and rotarod tests, as well as improved movement initiation and stepping time [89]. Recent in vitro and in vivo studies have also demonstrated that β-asarone treatment can protect PC12 cells and cortical neurons by inhibiting neuronal apoptosis through the activation of the calcium/calmodulin-dependent protein kinase II (CaMKII-α)/CREB/Bcl-2 pathway [90]. An in vivo study also indicated that β-asarone treatment improves cognitive function, reduces neuronal apoptosis, and significantly increases CaMKII/CREB/Bcl-2 expression in the cortices of APP/PS1 mice [90].

Myocyte-enhancing factor 2D (MEF2D) is a transcription factor that plays important roles in synaptic plasticity, neuronal development, and neuronal survival, and the dysregulation of MEF2D has been implicated in the pathogenesis of PD [178]. Increased MEF2D activity protected dopaminergic neurons from stress signals in rodent models of PD [179,180]. Enzyme-linked immunosorbent assays and immunohistochemistry analyses revealed that the β-asarone-mediated activation of MEF2D reduced the loss of TH-positive neurons in the mesencephalon, restoring nigrostriatal neuronal morphology in 6-OHDA-induced mouse models of PD [29], indicating that β-asarone can stimulate the activity of MEF2D via chaperone-mediated autophagy.

The growing body of evidence from both in vitro and in vivo studies indicates that α- and β-asarone exert neuroprotective effects by inducing NTF expression and/or potentiating NTF functions, besides their anti-apoptotic, and anti-inflammatory activities, and thus might have potential in the treatment of neurodegenerative diseases, such as AD and PD. However, additional preclinical and clinical studies are required to better understand these effects. Thorough in vivo studies examining the therapeutic potential of α- and β-asarone in other animal models of neuronal diseases, such as Huntington’s disease, cerebral ischaemia, schizophrenia, and other neurological disorders, are also still missing.

## 4. Neuroprotective Effects of α- and β-Asarone on Other Neurological Disorders

According to the WHO, approximately 10% of the global population suffer from depression and anxiety disorders [181]. Mitogen-activated protein kinases (MAPK) have been implicated in the pathophysiology of depression and addiction. A previous study reported that MAPK phosphatase-1 (MKP-1) is upregulated in CUMS-induced mice [182]. MKP-1 is not only expressed in response to stress but is also a key negative regulator of the ERK signalling pathway [183], contributing to depressive behaviour [184]. In the chronic CUMS-induced rat model, β-asarone treatment produced significant antidepressive effects [183]. Rats treated with β-asarone presented with significantly increased scores for horizontal and vertical activity in the open-field test and significantly increased total consumption of sucrose solution in the sucrose preference test, likely mediated by the inhibition of stress-induced hippocampal cell death. Furthermore, immunohistochemical and mRNA analyses indicated that β-asarone treatment protected hippocampal neurons from CUMS-induced cell death through the downregulation of MKP-1 [185].

In a study designed to investigate the antidepressant properties of essential oil and asarones from the rhizomes of *A. tatarinowii*, treatment with α- and β-asarone produced antidepressant-like activity, as measured by the forced swim test and tail suspension test [30]. Interestingly, α-asarone treatment was associated with a biphasic effect in the tail suspension test in mice, inducing an antidepressant effect at lower doses (15 and 20 mg/kg, i.p.) and a depressive-like effect at higher doses (≥50 mg/kg, i.p.) [186]. The antidepressant effect of α-asarone was mediated by its interaction with the noradrenergic (α1 and α2 adrenoceptors) and serotonergic (5-HT1A receptors) systems, whereas the depressive-like effects observed following the administration of high levels of α-asarone may be mediated by interactions with the GABAergic system [186]. In another study, α-asarone showed an anxiolytic effect in animal models of anxiety, as assessed by the elevated plus-maze, the light–dark transition test, the novel food consumption test, and the marble-burying test [187]. Treatment with α-asarone attenuated exogenous corticosterone-induced anxiety in rats, as assessed using the elevated plus-maze, hole-board, and open-field tests, by modulating the corticotrophin-releasing factor and neuroprotective cell signalling pathways [188].

The anticonvulsant effects of α-asaronol ((E)-3’-hydroxyasarone), an active constituent derived from α-asarone, were assessed in an in vivo anticonvulsant screening assay using the three most commonly employed mouse models of seizure, including 3-mercaptopropionic-acid (3-MP)-induced seizures, maximal-electroshock seizures (MES), and subcutaneous-injection-pentylenetetrazole (PTZ)-induced seizures [189]. The oral administration of α-asaronol resulted in a broad spectrum of anticonvulsant activity and displayed better protective indices (PI = 11.11 in MES mice and PI = 8.68 in PTZ mice) and lower acute toxicity (LD_50_ = 2940 mg/kg) than its metabolic parent compound (α-asarone). Additionally, α-asaronol displayed a prominent anticonvulsant profile, with a 50% effective dose (ED_50_) of 62.02 mg/kg in the MES model and an ED_50_ of 79.45 mg/kg in the PTZ model, whereas the control substance stiripentol displayed ED_50_ values of 240 mg/kg and 115 mg/kg, respectively [189].

## 5. Challenges to the Translation of the Neuroprotective Effects of α- and β-Asarone from Bench to Bedside

The pharmacological potential of α- and β-asarone has mostly been assessed pre-clinically. In fact, no clinical trial has yet been conducted to investigate the potential therapeutic benefits of α- and β-asarone in the treatment of neurological disorders.

One of the major issues limiting the clinical use of α- and β-asarone is related to their poor bioavailability. The oral bioavailability of α- and β-asarone is especially low because of their highly lipophilic character and thus poor water solubility. Pharmaceutical formulations of α- and β-asarone injection solutions typically require the use of solubility promoters such as Tween-80 or propylene glycol. However, these agents can cause allergic reactions [190,191]. To address this problem, alternative delivery systems have been developed. Drug delivery systems using liposomes and nanoparticles as carriers have been used successfully to encapsulate α- and β-asarone and increase their bioavailability [54,192]. Interestingly, the recent development of a novel α-asarone-embedded lipid nanoformulation has led to enhanced BBB transmigration and significantly increased α-asarone levels in murine plasma and inside the brain [54]. Nose-to-brain delivery via olfactory pathways is another unconventional method by which phytochemicals and other agents could enter the brain bypassing the BBB [193]. In this context, the intranasal delivery of α-asarone to the brain using mPEG-PLA and PLA nanoparticles has been shown to be a better brain-targeting strategy compared to i.v. administration. In addition, nasal-mediated brain delivery reduced liver accumulation and decreased drug-induced hepatotoxicity in rats [55,56]. Therefore, novel pharmaceutical delivery strategies may overcome the bioavailability issues of α-asarone, enhancing its potential effectiveness in humans. In the case of β-asarone, further studies on the most effective delivery systems need to be performed, not only to validate their efficacy but also to address potential safety issues.

Another important factor to consider is the dose-dependency of the pharmacological effects of α- and β-asarone. While α- and β-asarone at lower doses (<50 mg/kg) exhibit a wide range of therapeutic activities such as antidepressant, antianxiety, anti-Alzheimer, and anti-Parkinson effects, higher doses (≥50 mg/kg) result in hypomotility (decreased locomotor activity), and impaired motor coordination [27]. Therefore, further animal studies are required for understanding the toxicology of asarones in neurodegenerative disease models in order to establish a theoretical basis for developing safe α- and β-asarone-based therapeutics.

Importantly, toxicological studies have revealed that both α- and β-asarone can cause hepatomas and might possess mutagenic, genotoxic, carcinogenic, and teratogenic effects [27,64]. According to the European Commission [62] and the Joint FAO/WHO Expert Committee on Food Additives (JECFA) [194], β-asarone is not recommended for clinical use because of its dose-dependent toxicity, and thus the inability to establish a safe exposure limit. The development of more efficient systems to deliver α- and β-asarone to their biological targets, particularly the brain, might allow for effective localized biological action while minimizing toxicity. We strongly suggest that the bioavailability, biodistribution, tolerances, safety, and effectiveness of α- and β-asarone and their vehicles should be carefully addressed and taken into account when designing clinical trials, as these are common causes of trial failure.

The most important issue when targeting neurodegenerative diseases is bioavailability, particularly the ability to cross the BBB. Efficacy data provided by a number of preliminary studies have placed inappropriate emphasis on α- and β-asarone properties and provided little information about their bioavailability. More detailed studies are necessary to ensure that pre-clinical findings are more closely scrutinised in order to increase the likelihood of success of eventual clinical trials.

## 6. Conclusions and Future Prospects

α- and β-asarone display a range of pharmacological attributes that contribute to their protective effects against multiple neurotoxic stimuli. The neuroprotective effects of α- and β-asarone have been attributed to several potential mechanisms of action, including: (1) antioxidant properties; (2) the regulation of various neuroprotective signalling pathways; (3) the reduction of aggregate formation and promotion of the clearance of pathogenic protein aggregates; (4) anti-inflammatory properties; (5) the inhibition of microglial activation; (6) the activation of NTFs-mediated neuroprotection; and (7) the modulation of neurotransmitter levels associated with behavioural functions and neuronal cell survival. These neuroprotective features make α- and β-asarone potentially promising therapeutic agents for the treatment of various neurological disorders, including AD, PD, cerebral ischemia, and epilepsy. However, despite promising pre-clinical evidence, the successful translation of results from the bench to the clinic has yet to be achieved, due to the potential toxicity of α- and β-asarone and their low bioavailability. The development of new delivery systems might reduce α- and β-asarone toxicity and ultimately bring tangible therapeutic benefits for patients.

## Abbreviations

AChE, acetylcholinesterase; AD, Alzheimer’s disease; ADME, absorption, distribution, metabolism, and excretion; Aβ, Amyloid beta; APP, amyloid precursor protein; BBB, blood-brain barrier; BDNF, brain-derived neurotrophic factor; CAT, catalase; COMT, catechol-O-methyltransferase; COX-2, cyclooxygenase-2; CREB, cAMP-response element-binding protein; CUMS, chronic unpredictable mild stress; DA, dopamine; DOPAC, 3,4-dihydroxyphenylacetic acid; DPPH, α-diphenyl-β-picrylhydrazyl; ER, endoplasmic reticulum; ERK, extracellular signal-regulated kinase; GDNF, glial cell-derived neurotrophic factor; GR, glutathione reductase; GSH, glutathione; HD, Huntington’s disease; HPLC, High Performance Liquid Chromatography; HO-1, heme oxygenase-1; HSP70, heat-shock protein 70; HVA, homovanillic acid; IL-1β, interleukin-1β; IL-6, interleukin 6; iNOS, inducible nitric oxide synthase; JNK, c-Jun N-terminal kinase; LB, Lewy bodies; LDH, lactate dehydrogenase; MAO, monoamine oxidase; MAPK, mitogen-activated protein kinase; MDA, malondialdehyde; MPTP, 1-methyl-4-phenyl-1,2,3,6-tetrahydropyridine; NGF, nerve growth factor; NMDA, N-methyl-D-aspartate; Nrf2, nuclear factor-erythroid factor 2-related factor 2; NTFs, neurotrophic factors; NF-κB, nuclear factor kappa B; PD, Parkinson’s disease; PI3K/Akt, phosphatidylinositol-3-kinase; RNS, reactive nitrogen species; ROS, reactive oxygen species; SNpc, substantia nigra pars compacta; SOD, superoxide dismutase; TNF-α, tumour necrosis factor; TrkB, tropomyosin-related kinase B; UPR, unfolded protein response; UPS, ubiquitin-proteasome system; WHO, World Health Organization; 5-HT, 5-hydroxytryptamine; 6-OHDA, 6-hydroxydopamine.
